# Translating Family-Focused Prevention Science into Public Health Impact: Illustrations from Partnership-Based Research

**Published:** 2011

**Authors:** Richard L. Spoth, Lisa M. Schainker, Susanne Hiller-Sturmhöefel

**Keywords:** Underage drinking, child, adolescent, problem behavior, prevention, preventive intervention, family-focused preventive intervention, community-university partnership, evaluation, effective prevention strategy, information transfer from research to practice

## Abstract

Underage drinking is a pervasive problem in the United States, with serious consequences for youth, families, communities, and society as a whole. Family-focused preventive interventions for children and adolescents have shown potential for reducing underage drinking and other problem behaviors. Research findings indicate that clear advances have been made, in terms of both the number of evidence-based interventions available, and in the quality of the methods used to evaluate them. To fully reap the benefits of such preventive interventions and achieve public health impact, the findings of family-focused preventive intervention science must be translated into real-world, community practices. This type of translation can be enhanced through four sets of translational impact factors—effectiveness of interventions, extensiveness of their population coverage, efficiency of interventions, and engagement of eligible populations, with sustained quality intervention implementation. Findings from studies conducted by researchers at the Partnerships in Prevention Science Institute and other empirical work highlight the importance of these factors. A model for community–university partnerships has been developed that potentially can facilitate the dissemination and public health impact of universal interventions to prevent underage drinking and other problem behaviors. This model fits well within a comprehensive strategic framework for promoting effective prevention.

Underage drinking is a serious public health concern that places an enormous burden on affected youth, families, communities, and society as a whole. The pervasiveness of the problem is illustrated by findings from the Monitoring the Future Survey (Johnston et al. 2010), showing that even among 8th graders, about 15 percent had consumed alcohol in the month preceding the survey; this increased to almost 45 percent among 12th graders (see table 1). Furthermore, a significant proportion of the youth surveyed reported that they had been drunk in the month preceding the survey.

In addition to being illegal, underage drinking is especially worrisome because it can have a long-term or, in some cases, lifelong impact on an adolescent’s physical and intellectual development. For example, alcohol consumption might adversely affect the still-developing brain, causing potentially lasting changes in brain structure and function that are likely to negatively influence the individual into adulthood (Tapert and Schweinsburg 2006; Tapert et al. 2008). Also, adolescents who indulge in heavy drinking are likely to engage in risky behaviors, such as drinking and driving; traffic accidents pose the single greatest mortality risk associated with underage drinking (Grunbaum et al. 2002; Hingson and Kenkel 2004; Hingson et al. 2005). Likewise, alcohol-related risky sexual behavior (e.g., unprotected sexual activity) can lead to consequences such as sexually transmitted diseases and pregnancy (Grunbaum et al. 2002; Hingson et al. 2004; National Institute on Alcohol Abuse and Alcoholism [NIAAA] 1993). Moreover, adolescents who drink alcohol are at increased risk for behavioral problems, such as delinquency, violence, and poor academic performance (Hingson et al. 2002; Substance Abuse and Mental Health Services Administration [SAMHSA] 2008) and mental health problems, such as depression and suicidality (NIAAA 1997; Swahn et al. 2008; Windle and Windle 2001). Finally, underage drinking increases the risk for using other drugs during late adolescence and into adulthood (Ellickson et al. 2003) as well as for developing alcohol use disorders (AUDs)—that is, alcohol abuse and dependence—during adulthood (Dawson et al. 2008; Grant and Dawson 1997). In addition, these consequences of underage drinking result in substantial economic costs, which have been estimated to be approximately $62 billion per year (Foster et al. 2003; Levy et al. 1999).

Studies on the etiology of adolescent problem behaviors such as underage drinking indicate that such problems are influenced to a large extent by family factors. These influences can both increase the risk of problem behaviors and protect against the development of such behaviors. Thus, a family history of AUDs or certain parenting behaviors (e.g., inconsistent or harsh discipline) can increase a child’s risk of early alcohol use and later development of AUDs (Hussong et al. 2008; Latendresse et al. 2008). At the same time, family factors can reduce the likelihood that an adolescent will experience alcohol-related problems. Most importantly, an effective, positive parent–child relationship—characterized by child monitoring, parental involvement in the child’s day-to-day activities, and parent–child bonding or affective quality—provides a scaffold that helps children and adolescents develop the adaptive skills (e.g., self-regulation, emotion, and behavior) needed to protect themselves from underage alcohol and other drug (AOD) use (Elias et al. 1997; Masten and Coatsworth 1998; Mrazek and Haggerty 1994).

Because family influences are so pivotal in shaping adolescent problem behaviors, much research has centered on family-focused prevention approaches to reduce problem behaviors. For example, many well-designed studies have demonstrated that family-focused interventions (e.g., programs that focus on parenting practices, such as parent–child communication, parent–child bonding, and effective family management) can reduce problem behaviors in children and adolescents. Family-focused interventions can be successful both for general populations and for families with adolescents who exhibit more serious delinquent behavior (for a review, see Spoth et al. 2002*c*).

This article reviews the current state of family-focused prevention research and explores in more detail how these interventions can be translated from research projects to real-world settings. The authors summarize key findings from studies of various interventions and, as requested, focus on a program of partnership-based research at the Partnerships in Prevention Science Institute (PPSI), for illustrative purposes. They then discuss how the translation of existing and new interventions can be enhanced and how the translational impact of these interventions can be supported. This discussion primarily centers on a PPSI-developed model for community–university partnerships that focuses on the prevention of underage drinking and other problem behaviors, along with the national network that will support these partnerships.

## Moving Toward a Paradigm of Public Health Impact

To date, most of the family-focused interventions tested and proven to be effective only have been implemented with relatively small groups of adolescents and their families, either as part of a research project or as part of a small-scale dissemination effort. To fully reap the benefits of such preventive interventions and achieve a public health impact, it is necessary to translate the practices and findings of family-focused intervention science into real-world public health practices that can benefit large numbers of children, adolescents, and families. Therefore, it is essential that researchers, health care providers, relevant health services agencies, and policymakers adopt science-to-practice translational models oriented toward public health impact. Such models should ensure that programs and practices that are implemented on a large scale already have been proven to be effective (i.e., meet standards of evidence, such as those developed by the Society for Prevention Research [Flay et al. 2005]) and are implemented with sufficient quality on a sustained basis in community settings. These robust standards of evidence currently are met only by a limited number of programs and practices. In other words, although numerous family-focused interventions already are implemented in the United States, by far the majority of these interventions have not yet been rigorously evaluated. It is important to note that few interventions have demonstrated positive, long-term effects in rigorous studies, and fewer still are being implemented with sustained high quality (Spoth 2008).

Because of the limited large-scale dissemination and implementation of existing, evidence-based family-focused preventive interventions, it is critically important to pay close attention to specific factors influencing the translation of family-focused intervention research into large-scale, real-world applications. This requires that research, from the earliest developmental stages of an intervention onward, needs to take into consideration factors that ultimately could influence eventual large-scale implementation, such as consumer preferences. Even the most effective intervention likely cannot be implemented effectively on a large scale if the consumers (i.e., adolescents and their families) cannot be engaged sufficiently because, for example, the program requires too much of a time commitment.

## Current Status of Family-Focused Preventive Intervention Research

Although, as suggested above, it is clear that family-focused intervention can be of great benefit, the full potential of these interventions has not yet been realized. Nevertheless, over the past quarter century, researchers have made significant advances in family-focused and other types of prevention research—that is, in the development and rigorous evaluation of effective interventions.

Advances in the field of family-focused prevention research have been achieved across universal, selective, and indicated types of interventions (for reviews, see Alexander et al. 2000; Spoth 2008). (For a definition of the different types of interventions, see the text box.) Several advances are particularly noteworthy. First, universal interventions have shown long-term effects across a range of AOD misuse and related outcomes (e.g., health-risking sexual behavior or offending behaviors) for as long as 10 years past the baseline assessment of the outcome study (e.g., Spoth et al. 2009*c*). Long-term effects are mediated by delayed initiation of substance use, including underage drinking.

Second, selective interventions have been developed that easily can be integrated into Nationwide service programs, such as Head Start. Of importance, although these interventions necessarily initially are tested among relatively small populations, some of them have been developed from the outset with plans for subsequent, scaled-up implementation in large and diverse populations so that they can achieve a measurable public health impact.

Third, there is evidence of increased attention to cultural sensitivity of new programs, to ensure their applicability in different population subgroups. Finally, evaluation of family-focused preventive interventions increasingly has followed rigorous scientific standards, starting with intervention designs based on theory and continuing with outcome assessment using randomized, long-term studies with follow-up periods of at least several years.

Taken together, these advances have provided important information to researchers and clinicians alike, both on risk and protective factors for relevant problem behaviors among children and adolescents and on effective family-focused interventions aimed at preventing underage drinking and promoting positive youth development. However, although the potential for widespread dissemination of evidence-based family-focused interventions clearly exists, many challenging tasks remain.

### Review of Family-Focused Preventive Interventions

In a recent comprehensive literature review, Spoth and colleagues (2008*a*, 2009*a*) summarized the current state of the evidence regarding the effectiveness of all types of preventive interventions for underage drinking. The researchers identified more than 400 interventions that targeted different age groups (i.e., less than 10 years, 10 to 15 years, and 16 to more than 20 years) and were directed toward one or more of the different domains of the participants’ lives (e.g., school, family, or workplace). Of those interventions, 127 had sufficient information available to allow for an analysis of their effectiveness on the basis of six criteria (Spoth et al. 2008*a*).^1^ According to the extent to which these criteria were met, the researchers categorized the interventions into three groups:

- Interventions with the most promising evidence—these interventions met all six evaluation criteria, with the authors of the review making an overall judgment of how well the criteria were met;
- Interventions with mixed or emerging evidence—these interventions did not meet all six criteria but provided some evidence of effectiveness (e.g., they demonstrated a positive effect in some studies and no effect in other studies, demonstrated positive effects on some but not all measures, showed effects only in some subgroups of the sample, or demonstrated effects but had some substantial methodological limitations); and
- Interventions with insufficient or no evidence of effect—these included all interventions that did not fall into any of the preceding categories.

**Different Types of Preventive Interventions**

Depending on the target audience, interventions can be classified into three categories:

- Universal interventions are designed for all individuals in a given population (e.g., all middle-school students and their families in a given school district).
- Selective interventions are designed for specific population subgroups that as a whole are at higher risk of problem behaviors such as underage drinking (e.g., all students in a community who exhibit certain problem behaviors, such as antisocial behavior, and their families).
- Indicated interventions are aimed at specific individuals who have risk factors or conditions that place them at particularly high risk of a problem behavior such as underage drinking and related problems (e.g., adolescents who have been caught driving intoxicated).

Using this approach, a total of 12 interventions were classified as most promising and 29 interventions were classified as having mixed or emerging evidence (see table 2). The interventions with at least some evidence of effectiveness covered the entire range of included age groups as well as targeted family, school, and community or work place contexts. The analysis also supported the important role that family factors play in shaping children’s and adolescents’ behavior, especially among those ages 15 and under. At least 9 of 18 interventions aimed at children younger than 10 years of age and at least 5 of 13 interventions aimed at adolescents aged 10 to 15 years targeted the family domain. The following paragraphs briefly summarize some of the family-focused interventions and multicomponent interventions with a family component. For more detailed information on other preventive interventions for underage drinking, see the review by Spoth and colleagues (2008*a*).

#### Interventions for Children Under Age 10 With Most Promising Evidence

Few children under the age of 10 consume any alcohol. Therefore, interventions for this age group typically are designed to address other behavioral problems that often precede underage drinking (e.g., aggressiveness). By ameliorating these preceding problem behaviors, subsequent initiation of alcohol consumption can be prevented or least delayed. Two of the interventions directed toward children under the age of 10 that showed most promising evidence of effects on alcohol-related outcomes were specifically designed for parents or families. The remaining interventions were delivered as universal interventions to grade-school students and included both parent- or family-focused and school components. Each of the programs showed positive effects among the participating children. For example, the Linking the Interests of Families and Teachers intervention delivered in grade 1 led to reduced physical aggression in the children; in addition, when delivered to grade 5 students, it influenced alcohol-use patterns in middle school. Likewise, the Raising Healthy Children intervention resulted in less disruptive and aggressive behavior and later reductions in growth of alcohol use (although alcohol initiation rates did not decline). The Seattle Social Development Project demonstrated effects on aggression (at least in white boys), alcohol initiation in grade 5, and heavy drinking at age 18. A family-focused intervention for this age group, the Nurse Family Partnership Program, was designed specifically for low-income pregnant women. This program not only reduced mothers’ behavior problems attributable to AOD use but also resulted in fewer days of alcohol consumption among their offspring at age 15.

In summary, all of the programs for children younger than age 10 with the most promising evidence of effectiveness exclusively or centrally address the role of family-related factors in the development of problem behaviors (e.g., underage drinking and behaviors that precede it).

#### Interventions for Children Under Age 10 With Mixed or Emerging Evidence

Although most of the 13 interventions in this category included school-based strategies, some also included family-focused components, either alone (e.g., I Can Problem Solve) or in combination with school-based and other components (e.g., Families and Schools Together, Perry Preschool Program, The Incredible Years, Triple P-Positive Parenting). For example, the Families and Schools Together program involved 100 American Indian children of kindergarten and early grade school age; outcome research showed effects on aggression. Similarly, the I Can Problem Solve program involving 217 African American preschool-age children led to a reduction in impulsive behavior, which is considered a risk factor for early initiation of alcohol use. In the Perry Preschool Program, which was studied with 123 primarily African American preschoolers, the investigators noted reduced antisocial behavior at the follow-up assessments, although there were no differences in later adult alcohol use. The Incredible Years program was evaluated in three different samples of preschool-aged children. In each of these analyses, the researchers noted improvements on some measures but not on others, or the effects only were observed in subsamples of the children studied (e.g., those at higher risk). Finally, the Triple P-Positive Parenting intervention, which was evaluated with preschoolers in Germany and Australia, resulted in lower levels of externalizing behaviors (e.g., aggression and other problem behaviors).

#### Interventions for Adolescents Ages 10 to 15 With Most Promising Evidence

Early adolescence, with its transitions to middle and then high school, as well as the physiological and emotional changes brought on by puberty, is when many youth start experimenting with AODs. Epidemiological research has shown that the earlier an adolescent begins drinking alcohol, the greater is her or his likelihood of developing an AUD or other alcohol-related problems; therefore, interventions aimed at this age group might play a pivotal role in preventing alcohol initiation and its associated harmful consequences.

Several interventions have been developed that are directed toward this age group; many use a family-focused program, or a multicomponent approach that includes family components. One family-focused program in the “most promising evidence” category is the Strengthening Families Program: For Parents and Youth 10–14 (SFP 10–14). In one study, this intervention led to significantly lower rates of drinking and drunkenness at 4 years after baseline; moreover, alcohol initiation was delayed and lifetime alcohol use and drunkenness were significantly reduced at 6 years after baseline. More recent results from this study demonstrated that positive effects on reduction of alcohol-related problems extended into early adulthood (Spoth et al. 2009*c*). In a second study, the SFP 10–14 was implemented in combination with a school-based life skills training program. Findings from this study indicated that through 5.5 years past baseline, growth in alcohol and drunkenness initiation was significantly slower among adolescents receiving the intervention than among control adolescents, although not among a higher-risk subsample (Spoth et al. 2008*c*). These observations confirm that family-focused interventions can positively affect alcohol-related outcomes of adolescents at this particularly vulnerable age.

Two multicomponent interventions—Midwestern Prevention Project/Project Star and Project Northland—included family-focused components in addition to school-based and community-based components. These interventions also generated a variety of positive effects. For example, compared with adolescents from a control group, significantly fewer participants of Project Star reported past-week and past-month alcohol use. Among the participants of Project Northland, those who had received the intervention in grades 6 through 8 reported significantly lower past-week and past-month alcohol use, compared with controls, at 2.5 years after baseline, and those who received the intervention in grades 11 and 12 reported significantly less binge drinking at 6.5 years after baseline.

#### Interventions for Adolescents Ages 10 to 15 With Mixed or Emerging Evidence

Studies of two family-focused interventions for adolescents revealed mixed or emerging evidence of effects on alcohol-related outcomes. For example, the Family Matters program that was evaluated with adolescents from random households across the United States showed significant effects on lifetime alcohol use when the participants were assessed repeatedly; however, the size of the effects declined over time. Another intervention, Families That Care: Guiding Good Choices, reduced growth in alcohol use, past-month alcohol use, and past-month frequency of alcohol use at 4 years after baseline but did not significantly reduce past-year alcohol use. The New Beginnings program was developed for families that included 9- to 12-year-old children and their newly divorced custodial parents (mostly mothers); the intervention was directed either at both the mother and the children or only at the mother. Results from the outcome evaluation showed significant differences in past-year alcohol use frequency only among those families where only the mother had been targeted by the intervention; in that group, effects only were observed among participants with higher baseline alcohol use levels. Finally, an intervention called SODAS City, in which the content was delivered via a CD-ROM alone or in combination with a parent intervention, showed significantly lower past-month alcohol use at 3 years after baseline.

#### Conclusions

Taken together, review findings suggest that family-focused interventions can make a significant difference in children’s and adolescents’ lives, reducing their risk of underage drinking and its negative consequences. To date, many interventions have been administered to children 10 years of age or younger, when more of their time is spent with their families. They mainly focus on building healthy parent–child relationships, decreasing aggressive behavior, and strengthening the children’s social and cognitive competence for the transition into school. However, because of the young age of the children involved, the studies primarily assess risk factors that often are precursors of later alcohol use, such as aggressive behavior, rather than direct alcohol-related measures. Only studies with even longer follow-up periods can address whether these early interventions indeed reduce underage drinking, and particularly harmful drinking patterns, such as binge drinking. On the other hand, limited research has been conducted on interventions implemented during the “tween” years (later elementary-school years). Additional attention needs to be paid to the effects of family-focused interventions delivered during the middle-school years, when many young adolescents have their first experiences with alcohol. Finally, several family-focused interventions or multicomponent interventions with a family-focused component have shown evidence for reducing underage drinking and harmful drinking patterns even after extended follow-up, into young adulthood.

To transfer benefits demonstrated by the reviewed studies to the adolescent population at large, researchers now need to focus on translating these interventions into larger-scale implementation. The next section summarizes some of the recent advances in the translation of research on family-focused preventive interventions into effective, widespread application that achieves public health impact.

## Translating Family-Focused Intervention Research into Public Health Impact

**Figure 1:**
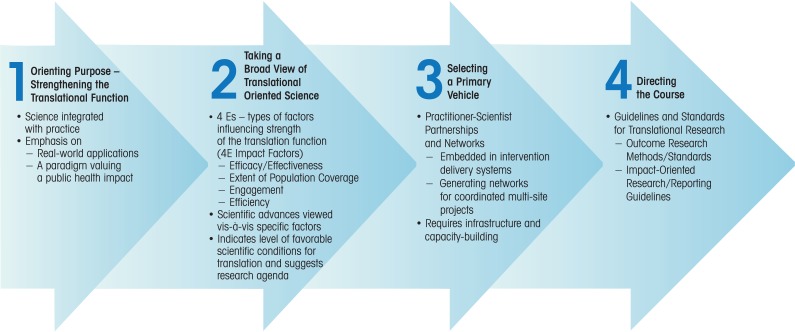
Overview of a strategy for plotting a course for public health impact through family-focused preventive intervention science. SOURCE: Adapted from Spoth 2008. Used with permission from Wiley-Blackwell Publishers.

For effective translation of evidence-based interventions into widespread practice that can have a real public health impact, four general steps can be helpful to consider (see figure 1). A first step is to enhance the translation of preventive interventions by considering not only the scientifically sound development and testing of the program but also considering, very early on, how organizational and systems factors specific to various practice settings ultimately will influence adoption, implementation, and sustainability of the program. A second step involves careful attention to specific sets of factors that influence the translation of interventions into widespread practice. These factors, also known as the “four Es of intervention impact,” will be described in more detail in the following sections. As a third step, mechanisms can be developed to facilitate the translation from research into practice—for example, practitioner–scientist partnerships and networks (these also will be discussed later in this article). Finally, a fourth step provides direction to the translational process by establishing appropriate guidelines and standards for translation-related research (e.g., standards on how intervention outcomes should be measured and how public health impact-oriented research should be reported and disseminated). Following all of these steps helps to ensure that effective family-focused interventions are developed and transferred into community practices that can have a positive impact on the future of adolescents and, consequently, a substantial public health impact.

### The Four Es of Intervention Impact: Illustrations From Partnership-Based Research

The following four factors are particularly important when considering the translation of preventive interventions, regardless of whether these interventions focus on the family or another contextual domain (Spoth 2008):

- The effectiveness of interventions;
- The extensiveness of their coverage of all populations potentially benefiting;
- The efficiency of interventions; and
- The engagement of populations and quality of intervention implementation with them.

The following sections address each of these factors in turn. Each section includes examples from extant research, many of which are illustrative examples from the authors’ program of research at the PPSI, as suggested for this article.

### The Effectiveness of the Intervention

The effectiveness of an intervention refers to the extent to which an intervention achieves a desired outcome, such as reduction in alcohol consumption, reduction in harmful drinking patterns, or reductions in related outcomes (e.g., alcohol-related traffic accidents or injuries) and is the most obvious requirement for serving the translation function and public health impact of preventive interventions.

Establishing effectiveness requires well-designed, methodologically sound studies that demonstrate practically significant outcomes, showing that the intervention can be considered to be “evidence based” (e.g., as per the outcome evaluation criteria described earlier). The results optimally can be replicated in independent studies. Moreover, researchers need to monitor and establish the long-term effects of the intervention, particularly when the desired effect (e.g., reduction of underage drinking) will be achieved only at some point in the future (e.g., when the intervention occurs during primary school or early middle school) or is supposed to persist for extended periods of time (e.g., throughout an adolescent’s school years). Researchers also should determine the core components or key mechanisms of the intervention that primarily are responsible for the intervention’s effects. Finally, in the case of targeting general populations, the universality of the particular intervention’s effects—that is, that the effects are observed across subgroups of participants that vary in risk levels—needs to be demonstrated. Several studies of family-focused preventive interventions, including those conducted at the PPSI, illustrate how the effectiveness of interventions can be evaluated and supported.

#### Analysis of Long-Term Effects

Although relatively few studies of family-focused preventive interventions have demonstrated long-term effects (i.e., at least 2 or 3 years past intervention implementation), the review summarized earlier uncovered some key studies in that regard. For example, a long-term follow-up study of the SODAS City intervention, which is a universal CD-ROM–based curriculum that includes a parent component, showed positive effects 3 years past baseline (Schinke et al. 2004). The Nurse Family Partnership program (Olds 1998) also has demonstrated replicated, long-term effects and quality implementation. Multiple randomized controlled trials have shown positive longitudinal mother-and-child outcomes, including reductions in child abuse or neglect and fewer arrests for both mothers and their 15-year-old children (also see www.nursefamilypartnership.org).

Another set of illustrative studies of long-term intervention effects comes from several randomized controlled clinical trials conducted by researchers at the PPSI (e.g., see Spoth 2007 for an overview). The purpose of these trials was to investigate the long-term effects of the interventions on alcohol- and gateway substance–related outcomes in adolescents. The aim of one study (Spoth et al. 2004a) was to analyze the effects of two brief family-focused interventions—the seven-session Iowa Strengthening Families Program (ISFP) and the five-session Preparing for the Drug Free Years (PDFY) program, delivered when the participants were in the sixth grade—on AOD use initiation 6 years after the baseline assessment. Throughout the follow-up period, the investigators examined a variety of alcohol, tobacco, and other substance use measures. Both family-focused interventions were able to slow the growth of initiation of AOD use over a 6-year period, with greater or more widespread effects seen for the ISFP. In a subsequent analysis (Spoth et al. 2009c), the investigators examined whether the delayed AOD use initiation reduced problematic AOD use during young adulthood, about 10 years after baseline. To this end, the researchers evaluated self-reports of several young-adult AOD use frequency measures. The analysis indicated that the effects of the interventions on adolescent AOD initiation indirectly led to a significant reduction in the frequency of a number of these measures.

### Effects Across Subgroups Targeted by Particular

#### Universal Interventions

Another important aspect of the effectiveness of general population interventions and their potential for translation to population-level impact is the universality of effects—that is, whether a particular intervention can be applied to, and generate positive effects for, all members of the specific population targeted or whether it is more or less effective in certain population subgroups. This includes, for example, gender-specific subgroups (Mason et al. 2009) and subgroups defined on the basis of risk profiles, including having a family history or other risk factors that predispose an adolescent toward AOD use. The relevance of this translation factor can be illustrated by an analysis of data from the previously mentioned study of sixth graders receiving either the ISFP or PDFY program interventions (Spoth et al. 2006*b*). For this analysis, the investigators divided the participating families into a lower-risk and a higher-risk group, based on 10 risk-relevant measures (e.g., the parents’ marital status, household income and financial strain, or presence of other psychiatric problems in the mother, father, or child). The analysis indicated that both interventions had comparable effects on alcohol initiation and illicit drug initiation; moreover, these effects were independent of the risk status of the family (i.e., the interventions were equally effective for adolescents from lower-risk and higher-risk families). These findings support the universality of the two family-focused interventions studied.

Other analyses, however, have indicated that some differences in effect depend on certain risk-relevant factors (e.g., parent education or household income [see Spoth and Redmond 2002]). Specifically, some interventions in some studies seemed to be more effective for youth from higher-risk families than in youth from lower-risk families. Spoth and Redmond (2002) proposed two mechanisms that may account for the greater benefit to higher-risk families: First, if higher- and lower-risk families are grouped together for the intervention, higher-risk families might begin to model behaviors from the lower-risk families (e.g., effective communication and problem-solving skills), thereby improving their outcomes. Alternatively, lower-risk families might be more likely to already apply the skills targeted by the intervention so that their overall benefits from the intervention are smaller than those of higher-risk families that have not yet been using those skills.

In cases where an intervention is shown to be less effective for higher-risk participants, it is important to consider a redesign of the intervention to better tailor it to higher-risk participants. Researchers previously had speculated that many intervention approaches, particularly brief, universal interventions, most likely would have greater benefits for lower-risk adolescents than for higher-risk adolescents (Offord et al. 1998; Spoth et al. 2008*c*). Nonetheless, as noted above, several more recent studies have suggested that family-focused interventions can have the same or even greater benefits for higher-risk youth than for lower-risk youth (Spoth et al. 2006*b*, 2008*c*).

#### Analysis of Key Mechanisms of Effects

It is important not only to evaluate whether interventions produce a positive effect but to understand how they produce these positive outcomes. An approach called core-component analysis allows researchers to better understand which components produce effects through the application of mediation analyses. Conducting core component analyses of multicomponent interventions allows program developers to learn which components are the most efficacious and indicates where program implementers might receive the largest return for their investment. This is especially helpful because multi-component interventions often are difficult to implement and expensive to replicate beyond the original research project. In one study of a 2multicomponent intervention called Project Northland, researchers conducted a post hoc core-component analysis to determine which of the key components—a classroom curriculum, a peer leadership component, extracurricular activities, a parent-focused program, and a community activism component—were driving the positive overall intervention outcomes (Stigler et al. 2006). The results indicated that only three of the five intervention components had a significant impact on alcohol use, suggesting that the remaining components were not essential ingredients.

To date, only a limited number of studies has used mediation analyses to examine mechanisms of effects for universal family-focused interventions; however, one such study comes from the PPSI (Spoth et al. 2009*b*). In this study, the ISFP intervention was found to exert its effects by establishing a “protective shield” that reduced adolescents’ exposure to illicit drug use. The researchers hypothesized that a family-focused universal intervention during sixth grade would reduce the number of illicit drug exposures or opportunities an adolescent had to use illicit drugs, thus providing a type of protective shield effect. The reduced exposure to drug use, in turn, was predicted to lower subsequent lifetime illicit drug use. Repeated waves of interviews of the study participants confirmed that, compared with adolescents in a control group, adolescents who participated in the ISFP experienced less illicit drug exposure and were less likely to have initiated illicit drug use by the end of the study. These findings suggest a plausible mechanism through which the ISFP could reduce illicit drug use.^2^ This finding was extended and replicated in a subsequent study. Overall, although a few studies have examined the mechanisms of effects, further study of the factors that mediate family-focused intervention effects is needed.

### The Extensiveness of Intervention Coverage of Diverse Population Targets

Achieving broad population-level impact depends greatly on the availability of evidence-based interventions for a wide range of targeted populations, including population subgroups defined by developmental stage. For example, for adolescents growing up in rural versus urban areas or having different ethnic backgrounds with varying cultural traditions, the family may play a different role in shaping their behavior. Accordingly, to have a broad public health impact, interventions need to be developed, tested, and disseminated to a wide variety of population subgroups and should address otherwise underserved populations. Basically, extensiveness of intervention coverage addresses whether interventions have been designed, tested, and proven effective for all relevant population segments, across developmental stages (i.e., across ages 1 to 20), with suitability to varying cultural contexts and settings.

Research has demonstrated that interventions designed for a more general American population (i.e., white, middle class) may not be well received by cultural subgroups that do not relate to program materials or messages inconsistent with the values and beliefs of their community (Kumpfer et al. 2002). According to Gonzalez-Castro and colleagues (2004), when such a “mismatch” occurs, intervention efficacy, even with high levels of fidelity, becomes threatened. There currently is a need for more interventions proven to be culturally competent. Greater cultural competence could enhance public health impact by increasing the likelihood that community members will be more highly engaged in intervention activities.

In an effort to increase the extensiveness of coverage of a preventive intervention, researchers at the PPSI (Spoth et al. 2003) conducted a pilot study that tested an adapted version of the previously mentioned SFP 10–14 to make the program more appropriate, culturally sensitive, and welcoming to African-American families. This adaptation primarily focused on the presentation of the intervention (e.g., inclusion of African-American families in program materials and use of African-American facilitators) rather than on intervention content. In a study sample of 110 families, the intervention positively affected certain behaviors and skills in the adolescents who were targeted by the program, such as goal setting, stress management, and effective communication with parents, but not parenting skills. The participants’ positive reaction to and acceptance of the program showed that interventions used primarily in the majority population could be successfully adapted for use in minority populations. Most notably, this pilot research contributed to a subsequent program of research that developed the Strong African American Families program.

On the basis of developmental research and cognitive models of adolescent health risk behaviors in African-American families, the Strong African American Families program focuses on strengthening regulated, communicative parenting processes (Brody et al. 2006). This means that the program instructs parents about the following: how to be involved in their children’s lives and closely monitor their activities while providing high levels of emotional and practical support; how to clearly articulate their expectations regarding adolescents’ alcohol use and sexual behavior; and how to provide racial socialization.^3^ The investigators found that this program, when tested in rural African-American families, could enhance these culturally relevant parent–child interactions and lower initiation rates of high-risk behaviors (Brody et al. 2006).

### Efficiency of the Intervention

The term “efficiency” refers to the relationship between the costs involved in administering the intervention and the economic and other benefits resulting from the intervention. For example, given that two interventions generate the same beneficial effects, a brief, lower-cost intervention, such as a self-administered program, will have greater efficiency than a more extensive, higher-cost intervention, such as repeated counseling sessions with trained facilitators. In addition, interventions that generate additional effects not initially intended (i.e., crossover, nontargeted effects) are more efficient than interventions that produce only the intended effects.

Assessing economic benefits of interventions also provides some indication of possible efficiencies, including analyses of costs, cost-effectiveness, and benefit-to-cost ratios. To date, relatively few interventions have been evaluated with rigorous economic analyses of these types. The findings from such evaluations suggest that economic benefits vary considerably, ranging from very positive to negative. Among youth-focused prevention programs, analyses have demonstrated that several family-focused interventions—or multicomponent interventions including family-focused elements—designed to prevent adolescent AOD use (e.g., Project Northland, Project Star, Family Matters) can be cost effective and beneficial (Aos et al. 2004). In general, cost-effectiveness data can help administrators conduct comparative analyses of interventions to better clarify which ones could likely produce a given effect at the lowest cost.

PPSI performed cost effectiveness and benefit-to-cost analyses using data from the long-term study of the ISFP and PDFY programs mentioned previously (see Guyll et al. in press; Spoth et al. 2002*a*). Cost effectiveness was determined by estimating the costs of treatment to prevent one adolescent from subsequently developing an AUD. This cost was approximately $12,500 per case prevented for the ISFP and approximately $20,500 per case prevented for the PDFY program. Other analyses have indicated that preventing one case of AUD results in a lifetime benefit of approximately $120,000. Dividing this amount by the cost per case prevented yields the benefit-to-cost ratio. Using this approach, the investigators found that for the ISFP, each dollar invested yielded a benefit of $9.60, whereas for the PDFY program, each dollar invested resulted in a benefit of $5.85. These findings demonstrate that family-focused interventions that require relatively small administration costs can result in substantial cost savings to society by preventing or delaying the onset of AUDs.

Crossover effects also can increase an intervention’s efficiency. Family-focused preventive interventions often are designed to target a number of proximal outcomes that previously have been shown to predict the desired long-term outcomes of interest, commonly described as risk and protective factors. These proximal outcomes can include protective factors (e.g., effective parenting, parent–child communication, and general relationship quality) as well as youth skills, such as the ability to refuse offered substances. The focus of the intervention might be to reduce gateway substance use, but because it targets risk and protective factors common to a range of problem behaviors or positive developmental outcomes, the intervention also might produce additional benefits beyond the one(s) primarily intended. In addition, because many problem behaviors frequently co-occur, reducing one problem behavior, such as substance use, is likely to “cross over” to reduce other problem behaviors as well. At a minimum, types of substance use not specifically targeted can be reduced (Spoth 2008; Spoth et al. 2006*a*). For example, one study of the effectiveness of the ISFP focused not on the program’s effects on adolescent AOD use but on adolescent aggressive, hostile, and destructive behavior (Spoth et al. 2000). The investigators determined that the intervention reduced aggressiveness and hostility in the adolescents’ behavior towards their parents (particularly their mothers) as well as outside of the home setting. Likewise, the ISFP has been shown to have positive effects on adolescents’ engagement in school in grade 8, their academic success in grade 12, and internalizing symptoms (Spoth et al. 2008*b*; Trudeau et al. 2007). These nontargeted effects resulted from the program’s positive effects on the proximally targeted outcomes, such as enhanced parenting skills and reduced risk of underage AOD use.

### Engagement of Target Populations and Quality of Intervention Implementation

The best intervention cannot produce positive effects, particularly at the public health level, if it is not accepted by or engaging to the target populations. In addition to initially engaging target populations (e.g., recruiting families), it must be implemented sufficiently well to maintain engagement or retention and to produce expected outcomes. Of note, poor implementation quality, including low adherence to intervention protocols, can substantially reduce intervention impact (Derzon et al. 2005).

Examples of engagement include recruitment of eligible families into the intervention, active participation by families during intervention sessions or activities, and attendance or completion of the entire curriculum. Research has indicated that motivation to participate in an intervention among families in eligible general populations can be significantly influenced by parent and youth characteristics and current behaviors as well as by family preferences and beliefs (Heinrichs et al. 2005; Pettersson et al. 2009; Spoth and Redmond 2000). Spoth and Redmond (2000) found that sociodemographic factors (e.g., ethnicity, educational attainment, or age of parents and children) have relatively little influence on recruitment and retention in universal interventions. Some studies, however, have indicated that ethnicity and family status (single-parent versus dual-parent families) can influence participation levels (e.g., Bauman et al. 2001; Rohrbach et al. 1994; Williams et al. 1995). Moreover, parent gender seems to play a role in intervention engagement because mothers seem to be more inclined to participate in a program (Spoth and Redmond 2000).

Parents’ beliefs regarding child problem behavior (i.e., whether parents consider the child susceptible to teen problem behaviors and consider those problems to be severe) or the child’s actual level of problem behavior also influence engagement (Heinrichs et al. 2005). Many families consider common adolescent problem behaviors (e.g., regular smoking and drinking or sexual activity) quite serious, although they often think that their own children are at low risk for these behaviors. Not unexpectedly, families that consider these problems to be serious frequently think that programs addressing these problems can be beneficial and therefore are inclined to enroll in such programs (Spoth and Redmond 2000). In a study of the Family Matters curriculum, parents who believed that their adolescents would smoke in the future were more likely to participate in intervention activities whereas those who believed that their adolescents currently smoked were less likely to participate (Bauman et al. 2001).

It is important to note that most of the information currently available on family engagement in preventive interventions comes from research staff–based recruitment efforts. However, these findings may not generalize to community-based recruitment by community volunteers; the latter would be critically important for large-scale implementation of evidence-based interventions (Glasgow et al. 1999, 2003).

One possible approach to promoting sustained recruitment and retention of participants for family-centered interventions is through community–university partnerships, in which community agencies or volunteers primarily are responsible for recruitment of families but receive technical assistance from a university-based team. One study from the lead authors’ program of research found that this approach can result in relatively high recruitment rates compared with other community-based recruitment rates reported in the literature (Spoth et al. 2007*a*). Specific features of an intervention (e.g., meeting times and locations, program duration, or facilitator background) also can affect family engagement decisions—for example, scheduling problems can serve as an important barrier to family participation (Heinrichs et al. 2005). Researchers or organizations seeking to implement an intervention need to pay attention to these factors to ensure acceptance by their target population.

Intervention implementer skills and other factors that influence the active, ongoing engagement of participants reflect on the overall quality of intervention implementation. As noted earlier, implementation is especially important because it is associated with the magnitude of the effects of interventions; even in the case of interventions that have been shown to be highly efficacious, poor implementation quality can greatly diminish effects (Derzon et al. 2005; Durlak and DuPre 2008). A key indicator of implementation quality is the degree to which the intervention is administered consistently, with strong adherence to the original intervention protocol. For this reason, intervention implementation quality often is considered to be one of the most important factors in translational research models (Society for Prevention Research, 2007, www.preventionscience.org). Quality monitoring is particularly important when interventions are implemented under real-world conditions by diverse community-based organizations (Dzewaltowski et al 2004; Glasgow et al. 1999, 2003; Spoth et al. 2002*b*, 2007*b*).

## Translational Impact Through Community–University Partnerships and Networks

As the preceding sections have demonstrated, family-focused interventions have shown promise in preventing underage drinking and use of other substances—effects that can translate into significant health and economic benefits for the adolescents, their families and communities, and society as a whole. Moreover, a range of studies have demonstrated that research to date addresses a number of factors (e.g., effectiveness, efficacy, and engagement) required for a greater impact on public health. An important next step in more fully realizing the impact potential of these preventive interventions will be to ensure their widespread dissemination and sustained high-quality implementation. A promising approach to achieving this goal is the establishment of partnerships between community organizations and researchers as well as larger-scale networks of such partnerships.

Such partnerships can address specific public health objectives for family-focused interventions, such as reducing adolescent AOD use or conduct problems. Moreover, community–research partnerships can help address the needs of underserved populations. For example, rural areas with their small, widely scattered, and diverse populations present a challenge for prevention research and implementation of existing interventions (Spoth 2007). Effective collaboration between researchers and rural practitioners can help address these challenges and facilitate the effective dissemination and implementation of family-focused interventions. For these and other reasons, partnerships between researchers and communities are a central component of recommendations made by the National Research Council and Institute of Medicine (2009) for putting knowledge into practice in order to prevent youth problem behaviors.

The need for such partnerships was supported by a seminal analysis by Hallfors and colleagues (2002) who evaluated the effectiveness of community coalitions to prevent AOD abuse. These coalitions were organized at the grassroots level, bringing together diverse groups and agencies to provide community education and awareness, prevention, and treatment. Analyses reported by Hallfors and colleagues (2002) showed no positive effects of these coalitions on adolescent AOD abuse, although a subsequent analysis by Hingston and colleagues (2005) found reductions in alcohol-related deaths in a subset of those communities. The investigators speculated that presence of many competing agendas and goals, lack of requirements to use tested and effective programs, and poor organization and implementation may have contributed to the very limited positive outcomes observed.

Although the concept of community–research partnerships seems logical, their actual implementation can be challenging, attributable in part to the different goals and methods typically used by researchers and community practitioners. Researchers, for example, generally focus on basic intervention science, taking a cautious, step-by-step approach to developing new interventions and aiming to conduct carefully controlled, randomized trials to demonstrate efficacy before moving on to dissemination of the program to larger populations. In contrast, community practitioners naturally are interested in practical solutions, regardless of whether they have been thoroughly tested, that can be applied immediately to the pressing problems in their community. Moreover, community practitioners often want to adapt existing interventions to their local needs and circumstances, whereas researchers often want to conduct replication studies with strict adherence to existing protocols in order to obtain a more solid research base (Spoth and Greenberg 2005).

Several approaches have been suggested to resolve these discrepancies in goals and methodology. For example, researchers and community practitioners should identify their overlapping goals early in the collaborative process (Price and Behrens 2003; Wandersman 2003). In addition, an ongoing, active interaction between scientists and practitioners is necessary throughout the process (Spoth and Molgaard 1999). When all stakeholders in community– research partnerships are aware of and respond to challenges, such partnerships can be of great value to all involved (Weissberg and Greenberg 1998).

### The PROSPER Model As an Example for Community–University Partnerships and Partnership Networks

**Figure 2:**
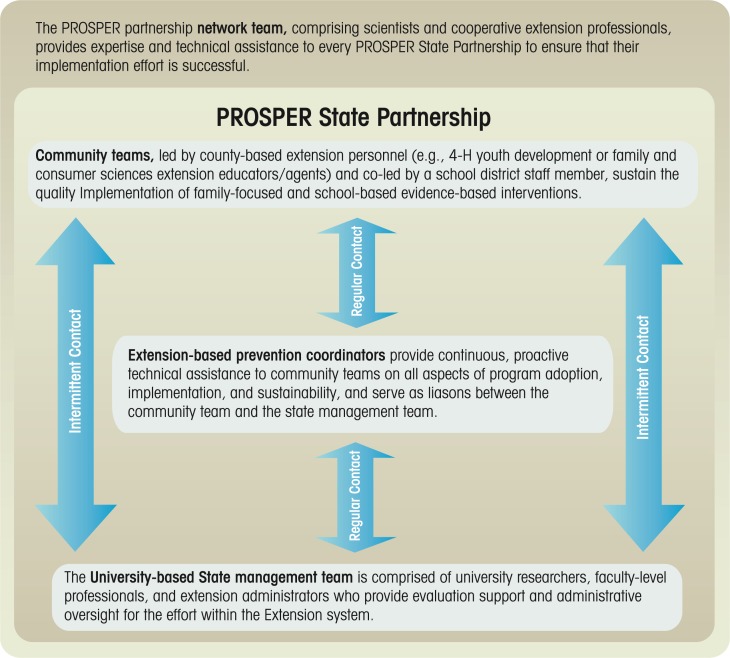
Conceptual diagram of the PROSPER partnership network model for the delivery of evidence-based interventions.

One partnership-based delivery system that strives to ensure effective translation of scientifically proven interventions to prevent underage drinking and AOD use into widespread practice is called PROSPER (PROmoting School–community–university Partnerships to Enhance Resilience). This system links university-based prevention researchers to two established program-delivery systems—the Cooperative Extension System (CES) at land-grant universities^4^ and the public school system (Spoth et al. 2004*b*). It evolved out of a program of partnership-based research over the past 20 years (Spoth 2007) that had produced many of the studies described above and was further developed by researchers at the PPSI at Iowa State University and the Prevention Research Center at Penn State University. It was specifically designed to test the effectiveness of a partnership that entailed small, strategic local teams as well as guidance from technical assistance partners, to facilitate sustained delivery of evidence-based programs (see figure 2).

PROSPER’s local teams are composed of three groups:

- County-level CES agents, who often are trained in community-leadership development and therefore can provide education and support;
- Elementary- and secondary-school representatives; and
- Local community service providers and other stakeholders (e.g., parent and youth representatives, law enforcement, or faith-based institutions).

These local community teams are in direct contact with an intermediate-level coordinating team consisting of CES-based prevention coordinators who provide direct technical assistance and administrative support, as well as act as liaisons with the university-based State management team. The State management team includes prevention scientists, CES specialists, and other collaborators and provides oversight and guidance, particularly concerning program implementation and data collection. The interaction of the community teams with the intermediate-level prevention coordinator team and, through it, with the State-level management team, ensures that the programs are implemented in the communities with sustained high quality.

Another key feature of the PROSPER model is that the preventive interventions used are evidence based. Moreover, the PROSPER model initially offers a menu of several such interventions (e.g., the family-focused SFP 10–14 and Guiding Good Choices interventions and the school-based All Stars Program, LifeSkills Training, and Project Alert) from which each community team can select the family-focused program and the school-based program that is most appealing or appropriate for their community. Eventually, the intervention menu is expanded. It is important to note that the researchers collect process data, allowing them to monitor how well the interventions are being implemented, as well as outcome data that address whether the interventions do indeed produce sustained positive results for the adolescents, their families, and their communities.

To date, the PROSPER model has been implemented in four States—Alabama, Iowa, Pennsylvania, and South Dakota. Work now is underway to expand it to an additional seven States. Results from the PROSPER model evaluation project have been very positive across a wide range of youth, family, and community outcomes, as follows:

- Numerous community teams successfully have been formed, progressed through the model’s developmental phases, and delivered family- and school-based interventions.
- Community teams have generated resources and sustained their programming efforts for up to 8 years.
- Community teams have consistently achieved relatively high recruitment and participation rates.
- Process data indicate that the interventions are implemented with high quality, with greater than 90 percent adherence to the intervention protocols.
- The programs implemented by PROSPER community teams have resulted in enhanced family strengthening, parenting skills, and youth skills. In addition, adolescents participating in the programs have lower rates of drunkenness, cigarette use, marijuana use, methamphetamine use, and other drug use, compared with the control groups, up to 4.5 years past baseline (see Spoth et al. 2007*c*; also Spoth and Greenberg, in press, and the PROSPER Partnership Group Research Overview for published papers available at: www.prosper.ppsi.iastate.edu).

These and other findings demonstrate that the PROSPER model is an effective approach for the prevention of underage drinking and other drug use, with the potential for Nationwide implementation. In response to interest from other States, a team of individuals from Iowa State and Penn State Universities are in the process of building the capacity and infrastructure necessary to support a network of PROSPER State partnerships. This network team, made up of prevention scientists and CES specialists from the original PROSPER model evaluation project, will serve as trainers and technical assistance providers to new State management teams and prevention coordinators as they develop and sustain their PROSPER State partnership.

Another community partnership model—Communities That Care—now has randomized controlled trial–based evidence of efficacy, although it does not specifically involve a partnership with universities (Hawkins et al. 2007). The Communities That Care system guides community-based coalitions through several activities, including the creation of a data-based community profile, development of a long-term action plan that includes selecting interventions that match community-identified priorities, finding resources to support their implementation, and evaluating their outcomes.

Regardless of which model of community-based intervention delivery is applied, it is critically important to create a comprehensive strategic framework for promoting and facilitating the spread of effective preventive interventions against underage drinking and other drug use that have proven translational capability to all regions of the Nation for maximum public health impact. Particular attention must be paid to capacity building of human, technical/scientific, financial, and other organizational resources, which is crucial for sustained quality implementation of any type of intervention. Three main tasks are involved in creating such a framework (Spoth and Greenberg 2005, in press). First, for any type of innovation to be effectively transmitted, “diffusion networks” must be established that facilitate the flow of information about the innovation. For example, the Public Health Service has stated that in order to effectively deliver interventions on a large scale, an adequate infrastructure, including data and information systems, must be in place (U.S. Department of Health and Human Services 2000). Second, an appropriate research agenda needs to be developed concerning evaluation of the larger-scale implementation of new interventions through community-based intervention-delivery systems. Relevant research questions include, for example, how complex community-based partnerships can best be evaluated, addressing implementation issues across diverse real-world settings. Third, it will be necessary to identify relevant policies needed for community partnership-based implementation of effective interventions on a large scale, such as policies regarding youth programming and community development, as well as economic policies to support such interventions. The Society for Prevention Research Task Force on Type 2 Translational Research is developing guidelines to foster a more strategic and systematic approach to such translational efforts (Society for Prevention Research 2007, available at: www.preventionscience.org).

## Conclusions

Family-focused interventions aimed primarily at preschool, primary-school, and middle-school students and their families have shown promise in preventing underage drinking and other related behavior problems. Children and youth participating in these interventions have shown improvements in behaviors commonly preceding underage drinking (e.g., aggressiveness), age at onset of drinking, frequency and amount of drinking, and other relevant measures. However, the development and testing of additional interventions clearly is needed. It is especially important that researchers consider developing new interventions focusing on what is required to enhance their widespread translation, so that interventions that yield positive results can be implemented on a large scale in real-world settings and not just under the confined conditions of a research trial. To demonstrate the translational capability of new and existing interventions, researchers need to address the four Es—Effectiveness, Extensiveness, Efficiency, and Engagement—of public health impact.

To maximize the translational impact of preventive interventions, it also is critical to assure sustained high-quality implementation of evidence-based, family-focused interventions on a large scale. Partnerships between practitioners in the community and university-based scientists offer a promising avenue. Such partnerships can offer community providers and organizations the technical support they need to build the capacities required to implement an intervention and to maintain high-quality implementation over a long period of time. At the same time, such partnerships can provide researchers with valuable feedback on what does or does not work in real-world settings. One successful example of such a community-university partnership is the PROSPER partnership model, which supports communities in implementing evidence-based family-focused and school-based interventions. Before the potential of such partnerships can be fully realized, however, a comprehensive strategic framework for expanding partnership networks, clarifying the necessary research agenda, and providing the necessary policy support needs to be developed. With the help of such a framework, effective interventions targeted at youth and their families can be implemented nationwide, reducing underage drinking and its harmful consequences, in order to achieve true public health impact.

## Figures and Tables

**Table 1:** Prevalence of Various Measures of Alcohol Use Among 8th, 10th, and 12th Graders in the United States in 2009 (Johnston et al. 2010)

**Measure of alcohol use**	**Percentage who reported alcohol use at some time in their lives**	**Percentage who reported alcohol use during the past 30 days**	**Percentage who reported being drunk in the past 30 days**
8th graders	36.6	14.9	5.4
10th graders	59.1	30.4	15.5
12th graders	72.3	43.5	27.4

**Table 2:** Interventions Designed for Different Age Groups of Adolescents With Some Level of Evidence of Effect and the Domains They Address*

**Level of Evidence**	**<10 Years of Age**	**Age Group 10 to15 Years of Age**	**16 to >20 Years of Age**
**Most Promising Evidence**	Linking the Interests of Families and Teachers (family, school) Raising Healthy Children (family, school) Seattle Social Development Project (family, school) Nurse-Family Partnership Program (family) Preventive Treatment Program– Montreal (multicomponent)	Keepin’ It REAL (school) Midwestern Prevention Project/Project STAR (multicomponent) Project Northland (multicomponent) Strengthening Families Program: For Parents and Youth 10–14 (family)	Project Toward No Drug Abuse (school) Yale Work and Family Stress Program (workplace) Mississippi Alcohol Safety Education Program and Added Brief Individual Intervention (community)
**Mixed or Emerging Evidence**	Classroom-Centered Intervention (school) Families and Schools Together (family, school) Fast Track (multicomponent) First Steps to Success (school) Good Behavior Game (school) I Can Problem Solve (family) Olweus Bullying Prevention (school) Perry Preschool Program (school, family) Promoting Alternative Thinking Strategies (school) Schools and Families Educating Children (multicomponent) Second Step (school) The Incredible Years (family, preschool, multicomponent) Triple-P-Positive Parenting (family)	Bicultural Competence Skills Program (clinic, school) Family Matters (family) Families That Care: Guiding Good Choices (family) (formerly Preparing for the Drug-Free Years) Healthy School and Drugs (school) Life Skills Training (school) New Beginnings Program (family) Project Alert (school) School Health and Alcohol Harm Reduction Project (school) SODAS City (family)	Athletes Training and Learning to Avoid Steroids (school) Brief Motivational Intervention in Emergency Department (community) Communities Mobilizing for Change on Alcohol (community) Community Trials Intervention to Reduce High-Risk Drinking (community) Problem Drinking in Workplace (workplace) Raising minimum drinking age law (State-level) Raising minimum drinking age law (school-level)

* For a description of the various interventions and their evidence, see Spoth and colleagues (2008*a*).

## Footnotes

1 These six criteria (see Spoth et al. 2008*a*) included the experimental design (i.e., was the study randomized or did it have a control group), sample specification (i.e., was sufficient information on the participants and their characteristics available), outcome assessment (i.e., did the study report outcome variables for at least three different time points), effects observed (i.e., were the differences in outcomes statistically significant), additional quality-of-evidence criteria (i.e., did the intervention meet quality-of-evidence criteria established by the National Registry of Evidence-Based Programs and Practices), and manualization (i.e., was a written manual available for the intervention).

2 Note that this study focused on illicit drug use and did not specifically assess alcohol-related outcomes; however, the mechanisms and factors that mediate impact on alcohol-related outcomes are likely to be the same.

3 The term “racial socialization” means that parents from minority groups (in this case, African Americans) must teach their children how to deal with a society that may devalue them and their families because of their race.

4 Land-grant universities are universities designated by each State that were established with funds raised from the sale or development of land given by the Federal government to the States in the late 19th century for this purpose. Part of the mission of these universities is to benefit the local communities. The CES was established in the early 20th century and is an informal education system designed to transfer research-based information to the general public. It involves thousands of local agents throughout the United States and, therefore, is well suited to disseminating prevention programs effectively.
